# Use of analytical strategies to understand spatial chemical variation in bacterial surface communities

**DOI:** 10.1128/jb.00402-24

**Published:** 2025-01-28

**Authors:** Abigail A. Weaver, Joshua D. Shrout

**Affiliations:** 1 Department of Civil and Environmental Engineering and Earth Sciences, University of Notre Dame 6111 , Notre Dame, Indiana, USA; 2 Department of Biological Sciences, University of Notre Dame 6111 , Notre Dame, Indiana, USA; Geisel School of Medicine at Dartmouth, Hanover, New Hampshire, USA

**Keywords:** *Pseudomonas aeruginosa*, quinolones, phenazines, rhamnolipids, Raman, mass spectrometry, chemical imaging, electrochemical imaging, biogeography

## Abstract

Not only do surface-growing microbes such as biofilms display specific traits compared to planktonic cells, but also they display many heterogeneous behaviors over many spatial and temporal contexts. While the application of molecular genetics tools to extract or visualize gene expression or regulatory function data is now common in studying surface growth, the use of analytical chemistry tools to visualize the spatiotemporal distribution of chemical products synthesized by these surface microbes is less common. Here, we review chemical imaging tools that have been used to inform our understanding of surface-growing microbes. We highlight the use of confocal Raman Microscopy, surface-enhanced Raman spectroscopy, matrix-assisted laser desorption/ionization, secondary ion mass spectrometry, desorption electrospray ionization, and electrochemical imaging that have been applied to assess two-dimensional chemical profiles of bacteria. We specifically discuss the use of these tools to study rhamnolipids, alkylquinolones, and phenazines of the bacterium *Pseudomonas aeruginosa*.

## INTRODUCTION

Surface-growing microbes such as biofilms display numerous aspects of biological, chemical, and physical heterogeneity (1–4). Even for a single species, surface growth manifests as a true assortment of “microclimates.” Not surprisingly, microbes experiencing varied microclimates can produce different biochemical products that further diversify the surrounding microbes over time and space.

**Fig 1:**
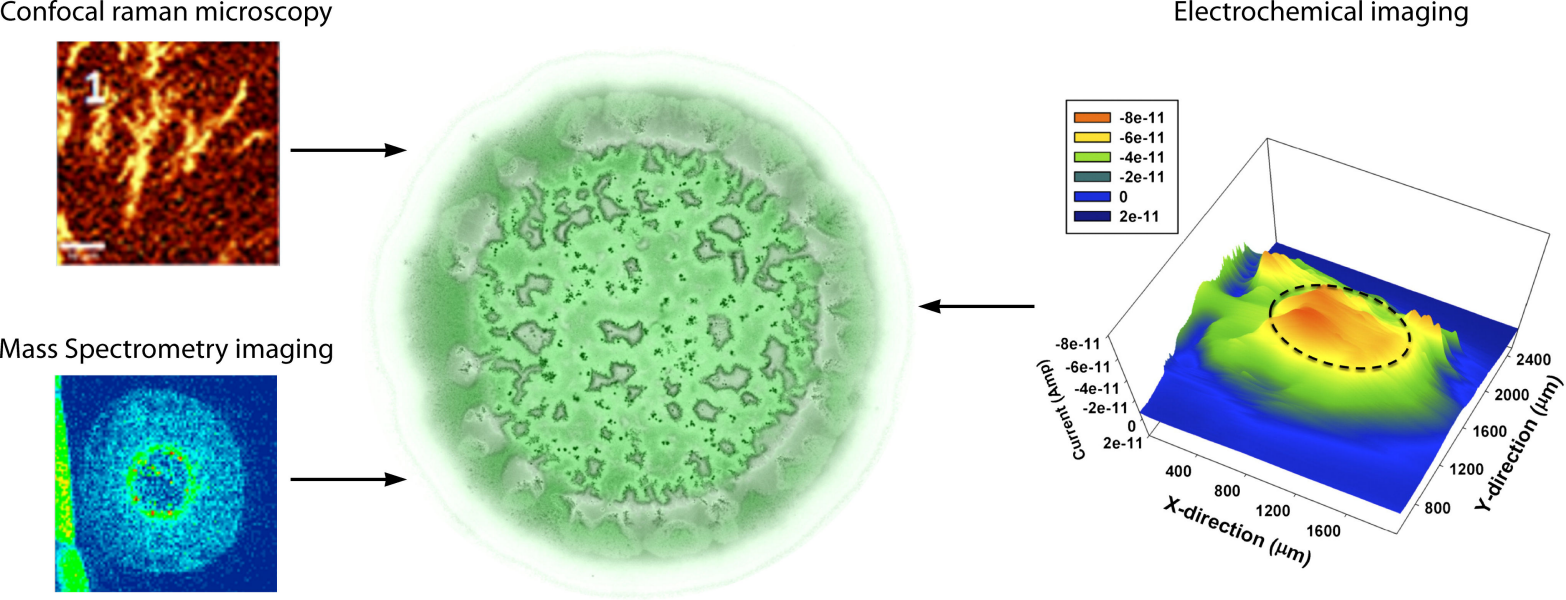
Spatial analytical methods for studying the distribution of compounds in *P. aeruginosa* biofilms.

Researching determinants of surface-growth heterogeneity requires more than a forward or reverse genetics approach. True functional insight necessitates knowing when and where microbial products are actually secreted and how these products influence the context of surface-growing communities. Increasingly, studies show the importance of biomolecular spatial heterogeneity and its impact on microbial fitness (2, 5). While familiar tools, such as fluorescence microscopy, continue to deepen our understanding of cellular biogeography (6–9), spatially detailed chemical information for surface-growing microbes is lacking. Most published studies provide only snapshots of the dynamic community organization that takes shape over time. Recent advances in spatial chemical imaging, such as confocal Raman microscopy (CRM), mass spectrometry imaging (MSI), and electrochemical imaging, are enabling increased discernment of biomolecular product heterogeneity (Fig. 1). Here we review analytical strategies that have been applied to the study of biochemical products important to bacterial surface growth and what we have learned from them (Table 1). Specifically, we describe products of the bacterium *Pseudomonas aeruginosa*, as a representative and well-studied microbe that is broadly relevant to many subfields. While the basic genetic elements for the examples we highlight in this review (rhamnolipids, alkylquinolones (AQs), and phenazines) are known, the complexity of their distribution in the two-dimensional (2D) and three-dimensional (3D) contexts of surface growth is marginally characterized, at best. In this regard, it is best to postulate that the full importance of these products remains to be elucidated.

**TABLE 1:** Methods applied to *P. aeruginosa* surface growth

Method^*d*^	Analytes	Experimental system/notable attributes	Disadvantages/current limitations	References
CRM	Alkyl-quinolones, EPS	No sample preparation^*a*^ Non-invasive and non-destructive^*a*^ Water does not interfere with analyte signal^*a*^	Not all molecules produce a detectable Raman signal.^*a*^ Molecules having the same Raman active vibrational modes can be indistinguishable.^*a*^ Weak signals can be masked in a sample.^*a*^ Fluorescence easily masks Raman signals.^*a*^ Depth of imaging is limited.^*a*^	Alkyl-quinolones (2, 10–15) EPS (16–18)
Surface-enhanced Raman spectroscopy	Phenazines	Signal of some molecular classes that exhibit weak Raman signal is increased 2–3×.	Requires the addition of metal nanoparticles Signal amplification is proximal to particles.	(19–22)
Stimulated Raman scattering	Phenazines	Enhances Raman signals	Requires expensive laser system Advanced setup focused on one vibrational energy of interest Analysis can be complex	(23)
Fourier transform infrared spectroscopy	EPS	No sample preparation Non-invasive and non-destructive	Has not been demonstrated with magnification like CRM for use at microbe–microbe level	n/a^*e*^
Mass spectrometry imaging–MALDI	Rhamnolipid congeners Alkyl-quinolones Rhamnolipids Phenazines	Requires matrix application to sample Can guide higher resolution imaging Maps a wide range of compounds Suitable for very large molecules	Destructive^*b*^ Limited to surface analysis^*b*^ Requires application of a matrix to the sample	(2, 24–28)
Mass spectrometry imaging–SIMS	Alkyl-quinolones	Greater spatial resolution than MALDI Fragments molecules Well suited to small molecules	Fragmentation makes it less suited to large-molecule detection.	(10, 12, 14, 25, 29–31)
Mass spectrometry imaging–DESI	Phenazines Rhamnolipids	Ambient sample analysis Little or no sample preparation identifies a large range of molecules	Molecule detection affected by ionization and solubility Matrix interference can reduce signal.	Phenazines (32) Rhamnolipids (33)
Electrochemical camera chip	Phenazines	Discriminates different phenazines	Requires fabrication of electrochemical camera chip	(34)
Scanning electrochemical microscopy	Phenazines	Real-time spatial imaging	Limited to electroactive compounds^*c*^ Can be non-specific^*c*^	(35–37)

^*a*^ Characteristic of all Raman imaging methods.

^*b*^ Characteristic of all mass spectrometry imaging methods.

^*c*^ Characteristic of all electrochemical imaging methods.

^*d*^ CRM, confocal Raman microscopy; DESI, desorption electrospray ionization; EPS, extracellular polymeric substance; MALDI, matrix-assisted laser desorption/ionization; SIMS, secondary ion mass spectrometry.

^*e*^ n/a: not applicable.

*P. aeruginosa* is a ubiquitous environmental organism that is also an opportunistic human pathogen (38). Transcription and production of many *P. aeruginosa* products are synchronized by quorum sensing. This review will focus on rhamnolipids, alkylquinolones, and phenazines. Rhamnolipids and phenazines are synthesized under the control of quorum-sensing regulons in *P. aeruginosa* (39–42). Synthesis of alkylquinolones does not require quorum-sensing induction (43, 44), but *Pseudomonas* quinolone signal (PQS) is a quorum-sensing signal molecule, and feedback loops are part of this regulon (45). While the examples we highlight here are specific to the pseudomonads and *P. aeruginosa*, many basic aspects of surface growth and biofilm development can be translated to understand how numerous other bacterial species differentially colonize surfaces.

## ANALYTICAL APPROACHES THAT HAVE BEEN USED TO STUDY SURFACE GROWTH OF *P. AERUGINOSA*

### Light spectroscopy

There are four variations of spectroscopic analytical approaches that have been used to image *P. aeruginosa* and other pseudomonad surface communities: Raman spectroscopy, surface-enhanced Raman spectroscopy (SERS), stimulated Raman scattering (SRS), and Fourier transform infrared spectroscopy (FTIR). Each of these spectroscopic methods relies upon elucidating chemical information by discerning emitted fingerprint characteristics upon illumination with targeted wavelengths of light.

Raman imaging distinguishes molecules through a categorization of atomic vibrational modes, measuring the inelastic light scattering of functional groups present in the sample. For microbial samples, application of Raman imaging has enhanced spatial resolution by employing confocal laser imaging and optical magnification to conduct CRM. With CRM, the spatial resolution of chemical information is on par with that achieved for light imaging—micron or submicron *x*,*y* discernment is realistic with high-magnification objectives of ≥×40. CRM is generally considered a non-destructive technology (equivalent to confocal laser scanning microscopy) in that the same sample can be imaged again. Alternatively, CRM can be used in tandem with a secondary analytical method, and the data can be reasonably spatially correlated (24, 29, 46).

One significant advantage of CRM over other spectroscopic or mass spectrometry techniques is that surface communities can be chemically imaged without the need for specific sample preparation. Microbial communities such as swarming colonies or biofilms grown on agar can be directly imaged, avoiding artifacts that might be introduced through manipulation of the sample before imaging. With CRM, three-dimensional images can be acquired, but reliable sampling depth does become limited by the ability of incident photons to penetrate through the biofilm (15).

Raman spectroscopy results are typically qualitative (or relative through comparison of samples), though quantitation has been achieved in some circumstances (47–49). This method is not suitable for all samples as not all molecules produce a Raman shift that is detectable in a “cell-silent” region where signals can be distinguished without overlap of shifts from a host of endogenous biomolecules from the cells (or the growth medium). Thus, Raman is optimally used for targeted approaches with a priori knowledge of a molecule’s diagnostic shift but poorly suited to resolve the question of “Which biomolecules are produced in greatest abundance in this sample?” Notably, water, which scatters weakly, does not interfere with the detection of target molecules. This lack of interference can make Raman a good choice for hydrated biological samples (50). There are instances where discrimination between different molecules that share the same Raman active vibrational modes is problematic. In such cases, a secondary analytical method is needed. Additionally, all Raman signals are inherently weak with only a fraction, typically 1 in 10 million photons, producing a signal (51), while with fluorescence, the quantum yield, the ratio of photons emitting fluorescence to incident photons, approaches 100% efficiency (52).

Modifications to Raman imaging have been developed to address weak signals. One technique that has been applied to *P. aeruginosa* biofilms is SERS (19–22, 53). SERS utilizes the localized surface plasmon resonance induced in noble metal nanoparticles, such as gold or silver, to enhance Raman scattering and improve signal. However, nanoparticles must be applied directly to the sample, making the method more complex and potentially invasive. Thus, fewer types of samples can be imaged using a SERS approach. SRS, which requires two synchronized lasers, termed the pump and Stokes beams, has a difference in frequency corresponding to a vibrational frequency of a molecule of interest. Upon interaction with the molecule of interest, the intensity of the pump beam decreases (stimulated Raman loss) and the intensity of the Stokes beam increases (stimulated Raman gain). It is the shift in these intensities that is measured. A drawback of SRS, however, is that detection is confined to a specific vibrational energy dictated by the experimental setup (51, 54, 55).

FTIR is another variation of light scattering imaging analysis. FTIR utilizes infrared light, specifically, as the excitation light. During FTIR, as with Raman, the spectral properties of light emission can be used to obtain chemical bond data of the analyte. The “Fourier transform” specifically refers to the reinterpretation of light waveform information to another format. FTIR has been most often applied to discern bacterial species or the presence of specific pseudomonad extracellular polymeric substance biopolymers by their amide I and/or amide II stretch regions (56–61).

### Mass spectrometry

MSI applies this technology to construct two-dimensional chemical heat maps of surfaces (25, 30, 62, 63). While mass spectrometry is a label-free technique that can identify a range of compounds, the ionization process is destructive and a portion of the sample is consumed during analysis. Advances in software have improved the process of deciphering the spatial analysis of MSI (64). However, quantification can be difficult due to challenges such as ionization efficiency, low concentration, or interference from matrices. The three components of a mass spectrometer, the ionization source, the mass analyzer, and the detector, work together to determine the mass-to-charge ratio (*m*/*z*) of ions. The molecules detected are heavily dependent on the ionization method, which also dictates spatial resolution and degree of sample preparation (65). Biofilm studies have been carried out using ionization methods that include matrix-assisted laser desorption/ionization (MALDI), secondary ion mass spectrometry (SIMS), and desorption electrospray ionization (DESI).

MALDI is a common technique that can achieve a spatial resolution of roughly 5 µm (65). It is worth noting that while pursuing increased spatial resolution and the ability to probe increasingly small areas is enabled, the number of biomolecules sampled can translate to results that fall below the level of detection. MALDI has been used with microbial samples to identify a broad range of analytes including metabolites, lipids, and proteins. The “matrix” of MALDI refers to a coating, such as 2,5-dihydroxybenzoic acid or α-cyano-4-hydroxycinnamic acid, that facilitates ionization and desorption of ions into the gas phase. Applying a matrix without disrupting delicate biological samples for spatial analysis can be challenging. Hydrated samples, such as agar plates, can absorb applied matrix which limits sample extraction, and analytes of interest can migrate during analysis. One option with such samples is to spray the matrix for a more even coating compared to a sieved matrix powder application (66). Alternatively, the matrix can be successfully applied after agar dehydration (67). While the agar shrinks, the morphology of bacterial colonies was found to be preserved (30). To improve resolving power, SIMS can be added, which uses a focused ion beam. Like MALDI, dehydration of the sample can reduce analyte migration, but no additional sample preparation is needed. Using MALDI followed by SIMS, a study showed that a lower resolution map can be constructed to guide a higher-resolution SIMS analysis (25). In the last method, DESI delivers solvent to a location in the sample, and the solvent/analyte is then desorbed from the surface under ambient conditions. As with other methods, analyte migration is reduced by drying samples. DESI, however, can be accomplished with no sample preparation and is carried out in the absence of a vacuum, allowing for ambient analysis of the sample (68). DESI is the most recently developed ionization source discussed here (69), and the majority of spatial studies applying DESI to microbial communities have focused on species other than *P. aeruginosa* (70–73).

### Electrochemical imaging

Of the molecules discussed here, phenazines are redox active and allow for electrochemical monitoring. Spatial imaging can be carried out using scanning electrochemical microscopy (SECM), in which a moveable ultramicroelectrode is able to measure local concentrations of redox-active molecules (34). Alternatively, a biofilm can be placed on an array of working electrodes for two-dimensional imaging (35, 36). Electrochemical sensors can be low cost and highly sensitive, though some can suffer from poor specificity (74).

## BIOCHEMICAL PRODUCTS STUDIED BY CHEMICAL IMAGING

### Example 1: rhamnolipids

Rhamnolipids are a variety of surfactant produced by strains of *Pseudomonas* and *Burkholderia*. Like synthetic surfactants such as sodium dodecyl sulfate, rhamnolipids lower surface tension and viscosity of the liquids in which they are dissolved. Diverse activities are associated with rhamnolipids including facilitating swarming motility and biofilm development, cytotoxicity against mammalian epithelial and immune cells, and increasing the bioavailability of hydrophobic molecules (including alkyl quinolones, discussed in the next section) (75–84).

**Fig 2:**
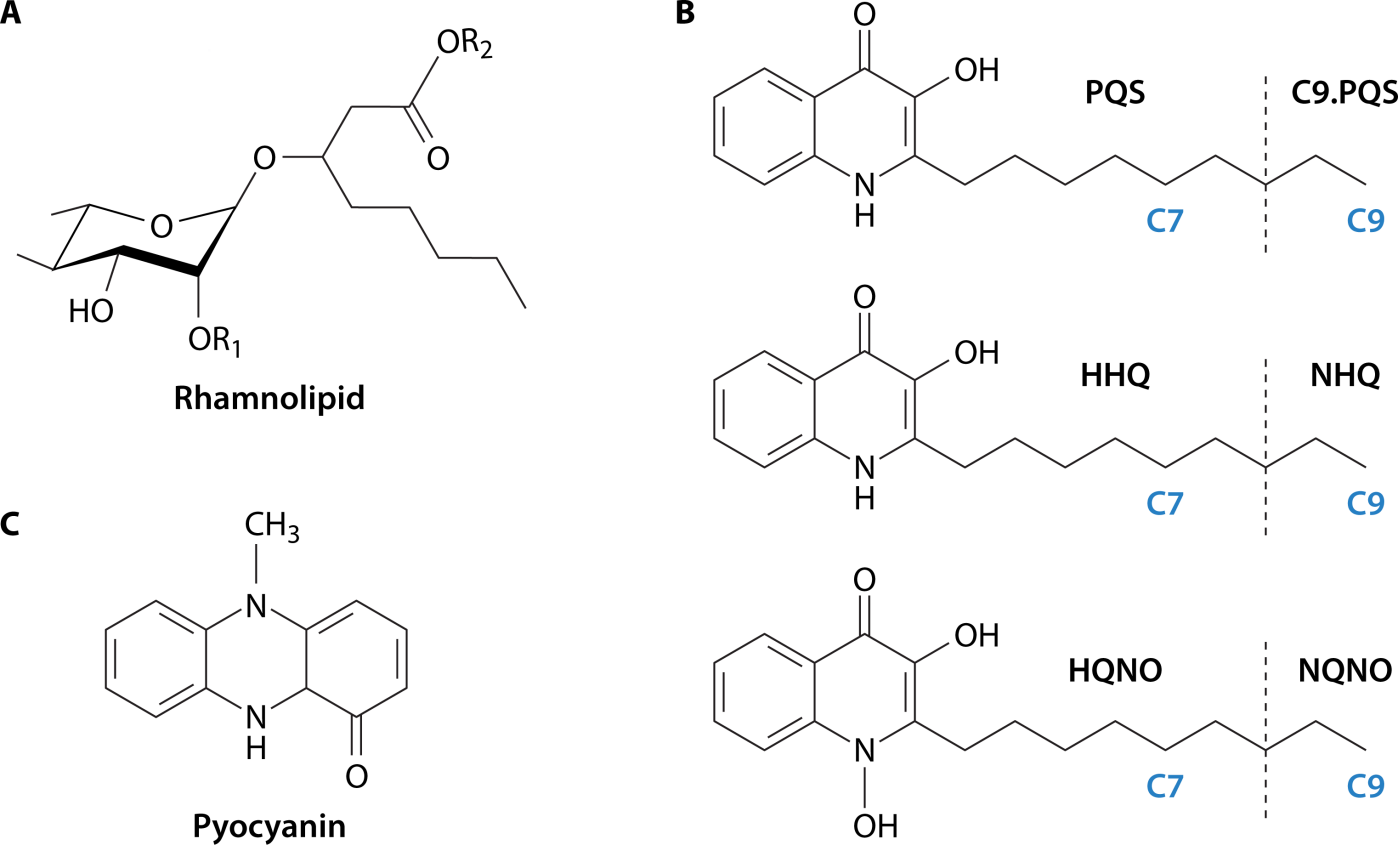
Chemical structures of *P. aeruginosa* secreted metabolites. (**A**) Mono-rhamnolipid where R1 is a hydroxyl or the site of an additional rhamnose to form a di-rhamnolipid, and R2 is a hydroxyl or the site for an additional acyl chain to form a di-acyl rhamnolipid. (**B**) Alkyl quinolones of the PQS pathway and (**C**) pyocyanin, a phenazine.

Rhamnolipid synthesis is enabled by three proteins: RhlA, RhlB, and RhlC. Transcription of the two-gene *rhlAB* operon is regulated under the Rhl quorum-sensing cascade. RhlA synthesizes 3-(3-hydroxyalkanoyloxy)alkanoic acid (HAA) from 3-hydroxy fatty acid precursors. HAAs also exhibit surfactant properties (85–87). RhlB and RhlC then act sequentially as RhlB adds a rhamnose moiety to HAAs to form mono-rhamnolipid and RhlC adds a second rhamnose to mono-rhamnolipid to form di-rhamnolipid (88, 89) (Fig. 2). As expected for gene expression regulated by quorum sensing, rhamnolipid gene expression is often observed to be highest in areas of greatest cell density. During swarming, expression is highest in the swarm center with decreased expression in the outermost tendril tips, suggesting an outward flow of rhamnolipids (90).

Because RhlA acts upon either acyl carrier protein–hydroxyfatty acids or coenzyme A–hydroxyfatty acids of multiple lengths to produce HAAs, *P. aeruginosa* can synthesize dozens of distinct rhamnolipids. In addition to variations in carbon chain length, use of some fatty acid precursors harboring carbon–carbon double bonds leads to a wide array of rhamnolipid congeners (89, 91). Congener names are abbreviated to reflect the number of rhamnose moieties, length and number of the carbon chains, and the presence of double bonds (92). While Zhu and Rock showed that 10-carbon chains are the dominant fatty acid precursors to be used by RhlA (87), any given strain produces multiple congeners. Overall, a wide range of rhamnolipids have been reported (85, 87, 93–95).

Most studies on *P. aeruginosa* rhamnolipid production and/or activity have grouped rhamnolipids by the presence or absence of RhlC, RhlB, or RhlA to group the corresponding products of di-rhamnolipids, mono-rhamnolipids, or HAAs, respectively. For example, on swarm assay agar surfaces, rhamnolipids were found to diffuse beyond the area of cell growth (26). Using disks impregnated with di-rhamnolipids or HAAs, studies have shown that the di-rhamnolipids diffused farther than their HAA precursors. This differential distribution was proposed to allow cooperative congener activity, both attracting (di-rhamnolipids) and repelling (HAAs) cells, to propel outward growth (96).

Morris et al. identified relative concentrations of dominant rhamnolipid congeners by analyzing entire swarm colonies using liquid chromatography–mass spectrometry (LC–MS) and found that swarm size correlated with changes in congener composition (94). However, a detailed spatiotemporal study of congener distribution in a *P. aeruginosa* swarm and how this impacts motility is still lacking. Certainly, many good questions have been postulated that require testing of strains that can produce only select congeners, but these tools do not yet exist. Nonetheless, some studies are beginning to shed light on the localization and behaviors of specific congeners in both two- and three-dimensional environments, though definitive models are yet to be identified to describe congener distribution within microbial communities.

Using MALDI-guided SIMS, where samples were collected spatially to allow reconstruction of 2D profiles, it was found that *P. aeruginosa* biofilms grown on solid surfaces submerged in liquid media are abundant in congeners Rha-Rha-C10-C10, Rha-C10-C10, and Rha-Rha-C10, with Rha-C8-C10, Rha-Rha-C10-C12, and Rha-C8 at lower abundance. In this fluid dominated environment, congeners displayed similar distribution patterns (25, 62). At the surface–air interface, however, congeners segregate, and complex distribution patterns emerge.

**Fig 3:**
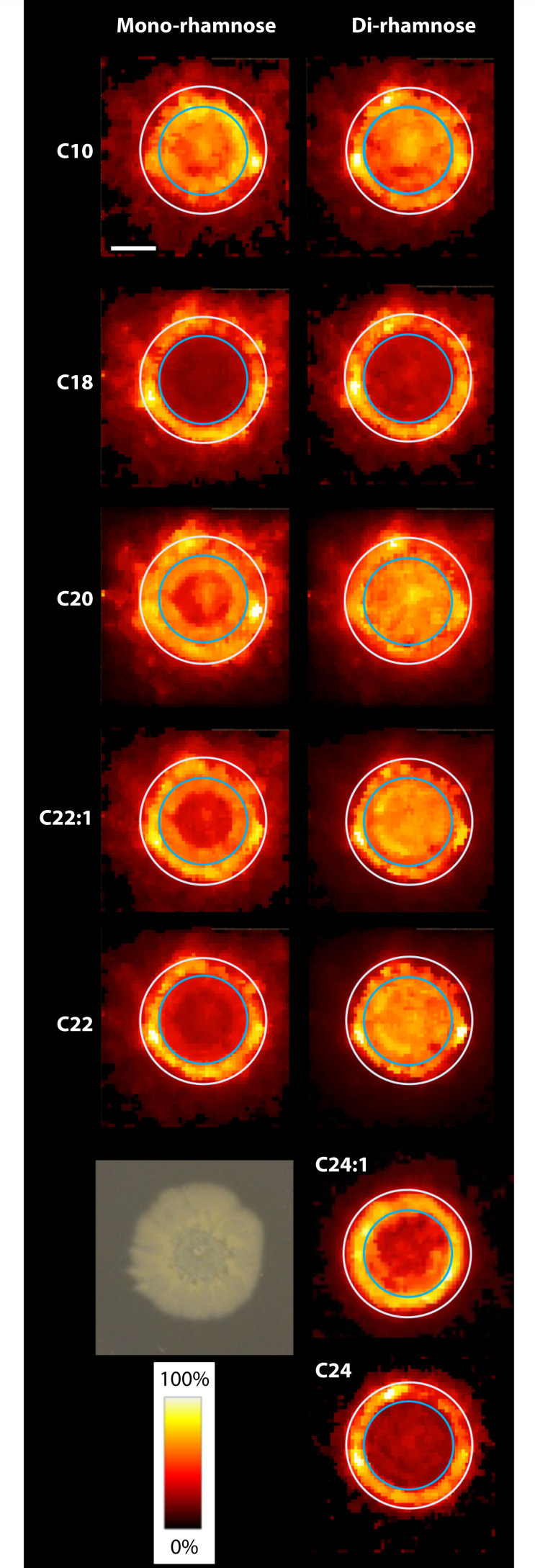
Distribution patterns and relative concentrations of rhamnolipid congeners in *P. aeruginosa* colony biofilms as determined by mass spectrometry imaging. Total carbons within acyl chains are designated in the left-hand margin. Reprinted from reference (2).

Initially, homogeneous mixtures of rhamnolipid congeners will separate from each other even in the absence of cells. In a subsequent study that also utilized MALDI (93), it was found that rhamnolipid congeners recovered from *P. aeruginosa* cultures as cell-free spent media then deposited onto agar have varying distributions with changes in fatty acid chain length. Interestingly, mono- and di- congeners with equivalent fatty acid chain lengths were most often found to have similar patterns of distribution, suggesting that earlier models in which HAA and di-rhamnolipids modulate motility may be oversimplified. Congeners with a total of 22–24 carbons in their fatty acid chain were more likely to distribute distally, forming a ring surrounding the deposition site, while those with shorter fatty acid chains were more centrally located. This pattern was replicated among some, though not all, commercial rhamnolipid extracts tested, showing that congener distribution patterns can be replicated, but that differences in the mixes, namely, the molecules present, shift congener distribution. Distribution patterns on agar alone, however, were not recapitulated within the dynamic environment of a growing colony biofilm. Here congeners exhibited more varied and complex patterns of distribution, with concentration shifts occurring between older and newer regions of growth (Fig. 3). Though previous studies identify trends within mono- or di-rhamnolipids, no unifying distribution patterns were produced by either group. Collectively, this suggests that the heterogeneity of rhamnolipid congener distribution within *P. aeruginosa* communities has been historically underestimated (93).

An even greater research challenge is to adequately describe a relevant *P. aeruginosa* growth environment. Garg et al. (97) studied distribution of rhamnolipids in cystic fibrosis lung carrying *P. aeruginosa* infection by tissue sectioning and extraction followed by ultra-high-performance liquid chromatography. While this approach obtains lower resolution than direct methods of chemical imaging, it is well suited to large sample areas (>1 cm). It was found that Rha-Rha-C10-C10 and Rha-Rha-C10-C12 rhamnolipids were readily abundant and found to have similar distributions in the lung. Curiously, these rhamnolipids were found with *P. aeruginosa* quinolones but not necessarily in regions where *P. aeruginosa* was directly detected (97).

We have known for years that environmental factors such as iron, nitrogen, phosphate, and carbon source availability impact bulk rhamnolipid production (91, 98–100). Now, application of analytical tools to study and map rhamnolipid spatial distribution is enabling new appreciation for the complexity of rhamnolipids. These most recent spatial studies highlight that our understanding of rhamnolipids is still in its infancy as the function of specific congeners that can separate away from regions containing high-density *P. aeruginosa* growth are poorly understood. These complicated distribution patterns are not easily modeled. Understanding how different single or combinations of rhamnolipid congeners contributes to *P. aeruginosa* survival, impacts upon other microbes and host cells, and affects congener gradients’ impact on local biomolecular bioavailability and flow is an area that requires further research.

### Example 2: alkylquinolones

Heterocyclic aromatic 2-alkyl-4(1*H*)-quinolones, commonly known as AQs, are a group of small molecules produced by select *Pseudomonas* and *Burkholderia* spp. Several AQs are known to act as antimicrobials or environmental signals at the species and interkingdom levels (101, 102). More than 50 alkylquinolones have been identified, and up to 39 have been simultaneously spatially imaged. However, the functions of most are not yet known (103, 104). There can also be modification of the produced AQs by other species that may be present in any given environment (105). These AQs can be divided into three subclasses: (i) 2-alkyl-4(1*H*)-quinolones, such as 2-heptyl-4(1*H*)-quinolone (HHQ) and 2-nonyl-4(1*H*)-quinolone (NHQ); (ii) 2-alkyl-3-hydroxy-4(1*H*)-quinolones, such as 2-heptyl-3-hydroxy-4(1*H*)-quinolone (Pseudomonas quinolone signal; PQS) and 2-heptyl-3-nonyl-4(1*H*)-quinolone (C9-PQS); and (iii) 2-alkyl-4-hydroxyquinoline *N*-oxides (AQNOs), such as 2-heptyl-4-hydroxyquinoline *N*-oxide (HQNO) and 2-nonyl-4-hydroxyquinoline *N*-oxide (NQNO)(103).

Production of the AQs in *P. aeruginosa* first requires amination of the versatile precursor chorismate to anthranilate. Anthranilate is then acted upon by PqsA and then PqsBC, PqsD, and PqsE to generate numerous 2-alkyl-4(1*H*)-quinolones. While PqsD appears to act on products of PqsA, consideration of multiple literature reports suggests that the activity order of PqsBC, PqsD, and PqsE is not strictly fixed, which leads to added product diversity. The PqsB,C acyltransferase activity adds chains containing either seven or nine carbon chains. Lastly, PqsL or PqsH act upon select 2-alkyl-4(1*H*)-quinolones to generate 2-alkyl-4-hydroxyquinoline *N*-oxides or 2-alkyl-3-hydroxy-4(1*H*)-quinolones, respectively (106).

There are four specific AQs that can act as quorum-sensing signals by binding to PqsR to upregulate genes of the PQS cascade (45, 107). These are HHQ and PQS and their nine-carbon equivalent molecules, NHQ and C9-PQS. Production of PQS and C9-PQS is unique to *P. aeruginosa* (108, 109) (Fig. 2).

PQS has several functions including chelating environmental iron (110, 111), regulating host immunity (112–114), promoting biofilm formation (115), and mediating cell death (116). PQS secretion has been shown to be mediated by packaging within membrane vesicles (117, 118). Additionally, both HHQ and PQS were found to significantly limit motility of some Gram-positive and Gram-negative bacteria, including *Staphylococcus aureus*, *Bacillus subtilus*, and *Vibrio fischeri*, in an iron-independent manner (119).

HQNO and NQNO are not known to be quorum-sensing signals. It is known that HQNO inhibits bacterial respiration and causes cell death and extracellular DNA (eDNA) release (120). It is secreted from cells and known to have antibiotic activity toward *Staphylococcus aureus* and other Gram-positive bacteria (121), providing an advantage in mixed microbial environments. Given this variety of roles, the localization of AQs in the biofilm can have a profound effect on regulating processes and providing a competitive edge in mixed microbial communities.

Early work identifying alkylquinolones was done in liquid culture (103, 122). In recent years, much progress has been made to map the temporal and spatial appearance of these compounds during growth on a surface in both biofilm and motile stages of *P. aeruginosa* growth. Analysis by SIMS compares liquid and surface-grown populations. In liquid samples grown in minimal media, many AQs fell below the limit of detection with only low levels of HHQ and NHQ detected. Similarly, colonies grown on solid media with abundant nutrients (lysogeny broth-Lennox [LB] medium) were found to contain low levels of AQs. However, cells grown on the surface of silicon wafers for 7 hours in liquid minimal media containing 12 mM glucose generally showed a 10- to 100-fold increase in AQs over liquid cultures and LB colonies (10). Reductions in glucose alone have been shown to result in increased PQS in surface-grown colonies (11). PQS is produced in response to specific stressors but not to generalized stress. Conditions such as phosphate limitation, competing bacterial strains, and some antibiotics increase PQS production (11, 12, 123), while the stringent response suppresses PQS and AQ production in general (124). In a surface environment, these stimuli occur in a localized fashion, diversifying the population in a way that is not possible with growth in liquid culture.

This varied biogeography was shown using MALDI-guided SIMS on silicon tiles at 72 hours. Lanni et al. found *P. aeruginosa* to have a heterogeneous distribution of AQs with microscopic pockets of PQS and HHQ. Contrastingly, a quorum-sensing double mutant (*ΔlasI*/*ΔrhlI*) produced HHQ throughout the biofilm with relatively lower amounts of PQS (25). In a mutant overproducing exopolysaccharides, Davies et al. found PQS to be clustered, while HHQ and NHQ were uniformly distributed (31).

In expanding swarms, AQs display even more distinct localization patterns. Using CRM, HHQ remained undetected, while PQS and HQNO exhibited distinct localization patterns. HQNO was produced by 24 hours at the edge and swarm center. This localization persisted at 48 hours when PQS also appears in the swarm center. In contrast, colony biofilms, grown at lower hydration levels, have both PQS and HQNO evident as early as 8 hours and remaining through 48 hours (13).

Exposure to some antibiotics can promote PQS production. Using CRM to study this response in swarming populations, Morales-Soto et al. found PQS (both C7- and C9-PQS) present proximal to tobramycin but not carbenicillin. SIMS was used to determine relative abundance of AQs in regions of the swarm. At 48 hours following exposure to tobramycin, higher levels of both PQS and HQNO were detected. The ratio of PQS to AQNOs increases significantly at the swarm edge closest to a 25 µg tobramycin exposure. This shift is not seen at a lower, minimally inhibitory level of 10 µg tobramycin (12). While carbenicillin and tobramycin are both found to inhibit swarm motility, their impact on the biomolecular landscape is quite different. Looking at a macrolide antibiotic, a combination of MALDI and liquid chromatography finds alkylquinolones, rhamnolipids, and phenazines, all discussed in this review, increase with increasing levels of azithromycin (26).

**Fig 4:**
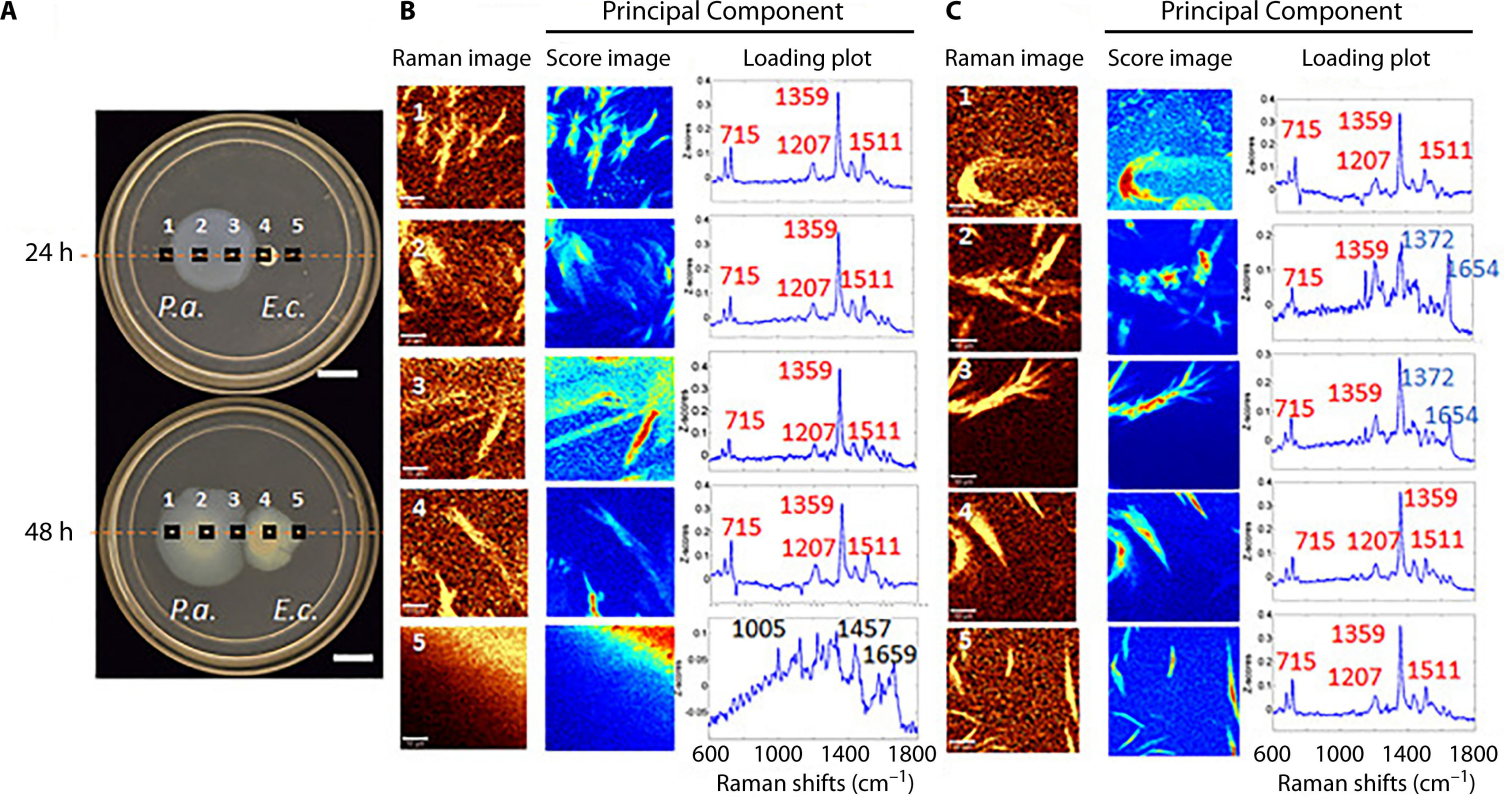
*P. aeruginosa* grown adjacent to *E. coli* at 24 and 48 hours. (**A**) Image of plate assays, (**B**) 24 hour associated Raman images, heat maps, and loading plots, identifying peaks characteristic of the alkylquinolones, HQNO (blue lettering) and PQS (red lettering), and (**C**) equivalent Raman imaging results from 48 hour time point. Reprinted from reference (11).

Investigating AQ shifts in response to competing microbes, Cao et al. (11) find that in colony biofilms, PQS appears 48 hours earlier when cultured in proximity to *Escherichia coli* K-12. Specifically, PQS production increases locally at the intersection of these two species but not broadly throughout the population (Fig. 4). This increase is not seen in response to a second *P. aeruginosa* colony. Contrastingly, HQNO does not exhibit differential expression either spatially or temporally with exposure to *E. coli*. However, it was noted to precede *P. aeruginosa* colony expansion and occupy space within the *E. coli* colony. Increased PQS production was also seen following exposure to cell-free cultured media from *E. coli*, indicating that cell-to-cell contact is not required to promote increased PQS production (11).

The PQS response was hastened with a reduction of glucose from 12 to 6 mM in minimal media, but at the lowest concentration of 3 mM glucose, no AQs were produced despite apparent colony growth and expansion (11). This supports that AQ production is responsive to changes in nutrient sources but has a metabolic threshold and is in agreement with studies supporting loss of AQ production during the stringent response (124).

To discriminate between AQ congeners, SIMS was applied, showing that C-9 forms of PQS/NQNO were in highest abundance in 7 hour biofilms with an increase in C-7 PQS/HQNO at 48 hours (10). The importance of the shift in dominance from one to another is yet to be understood but has the potential to fine-tune the function of these chemical messengers over time and space. The abundance of alkylquinolones with slight structural modifications has the potential to gain synergistic effects from two molecules. This was shown with HQNO and HMNQ in *Burkholderia thailandensis*. The two molecules inhibit the cytochrome bc1 complex by different mechanisms. At a 1:1 ratio, there is a synergistic increase in antibiotic effect, predominantly on Gram-positive microbes (125). Few 2D studies exist discriminating *Pseudomonas* secondary metabolite congeners, and little is known about their co-localization and ability to spatially and temporally tune activities.

Using CRM, Weaver et al. recently determined that AQs can be sequestered in aggregates within colony biofilms. Aggregates were associated with signals of HHQ/NHQ, C-7/C-9 PQS, and HQNO/NQNO while in regions outside of these aggregates AQ levels fell below the level of detection. This aggregation allows some cells to be exposed to AQs, while others are not. AQ-rich aggregates were associated with pockets of cell death in wild-type biofilms, while strains lacking the PQS pathway experienced increased, homogeneous levels of cell death. AQs were necessary for aggregate formation, identifying a role for alkylquinolones as drivers of biofilm heterogeneity. AQ-driven spatial organization appears to benefit the colony by promoting limited regions of cell death (93). Pockets of cell death supply surrounding cells with vital nutrients, provide DNA for genetic exchange and biofilm strengthening, and eliminate damaged cells (126, 127).

In studying the biofilm surface, both CRM and MSI have limitations in discriminating molecules. This limitation is apparent in the study of *P. aeruginosa* alkylquinolones. Alkylquinolones can vary by both functional group and length of acyl chain. For example, the C7-PQS and C9-PQS forms contain the same functional groups but vary in length of acyl chain (Fig. 2). These forms cannot be distinguished by CRM but are easily distinguishable by MSI through differences in *m*/*z*. However, MSI cannot distinguish between isomeric pairs such as C7-PQS and HQNO or C9-PQS and NQNO that differ in structure but have the same *m*/*z*. These molecules, however, are easily distinguished by tandem mass spectrometry, ion mobility mass spectrometry (128), or CRM (10), where there are means to distinguish the differences in functional groups. Combining MSI and CRM, a study has shown that these orthogonal approaches provide a powerful approach for deciphering the distribution of related molecular species present in bacterial communities growing on a surface (10). Both CRM and MSI have been carried out with a variety of microscopic techniques as well (i.e., brightfield, fluorescence, and polarized light), which also offer a workflow for gathering chemical information in two and three dimensions.

### Example 3: phenazines

Phenazines are three-ring, carbon–nitrogen heterocyclic compounds that are known to be synthesized by species of *Pseudomonas*, *Burkholdia*, and *Streptomyces* (129). Phenazines are made from chorismic acid precursors (130, 131). While several phenazines were originally identified and described because of their pigmentation (132, 133), these compounds are now also known for their ability to shuttle electrons, bind metals, act as antimicrobials, and increase gene expression related to transport (41, 129, 134, 135). The phenazine pyocyanin (PYO) is well studied and has been recognized as a hallmark of *P. aeruginosa* pathogenesis for well over a century as the critical component of cyan blue pus in wound infections (133). PYO is blue in its oxidized form, and production is driven by PQS quorum sensing or interaction of *pqsE* with *rhlR* (21, 42, 136–144). Pyocyanin has subsequently been determined to be an important component of *P. aeruginosa* lung and wound infections (145–148). At physiological pH, pyocyanin is a zwitterion that can cross biological membranes (149) and deplete the reduced form of glutathione, a powerful antioxidant, allowing the formation of harmful reactive oxygen species (150) and autolysis. In biofilms, this increases local eDNA accumulation and strengthens the biofilm (151). However, in oxygen-limited conditions, the phenazines PYO, phenazine-1-carboxylic acid (PCA), and 1-hydroxyphenazine can each promote cell survival by supporting redox cycling (152). Thus, the local environment of phenazines is key in determining their role in a biofilm.

Polisetti et al. find pyocyanin to have a heterogeneous distribution in pellicle biofilms using SERS. This was true of pellicles grown with glutamate as a carbon source but not with glucose, in which case PYO was not detected (21). The dependency of PYO levels on nutritional environment was also observed by Simoska et al. using DESI–MSI on colonies grown on trypic soy broth (TSB) or LB (thus, sodium chloride concentrations were equivalent). PYO concentration was lower with TSB compared to LB; however, the highest levels of PYO for both were in the outer regions of cell growth (32). Strain also determines levels of PYO as the mucoid stain FRD1, unlike wild type, produces detectable PYO with glucose, though the spatial distribution has not been reported (14, 21).

Evans et al. used SRS, a technique that enhances Raman signal, to probe metabolic shifts in the biofilm depth. In lieu of direct metabolite sensing, this work spatially measured deuterium incorporation into the biomass as a measure of metabolic activity. To probe biofilm depth, extensive sample prep and sectioning was required, yet, by this method, investigators were able to determine the factors allowing biofilms to limit the toxic effects of methylated phenazines (23).

**Fig 5:**
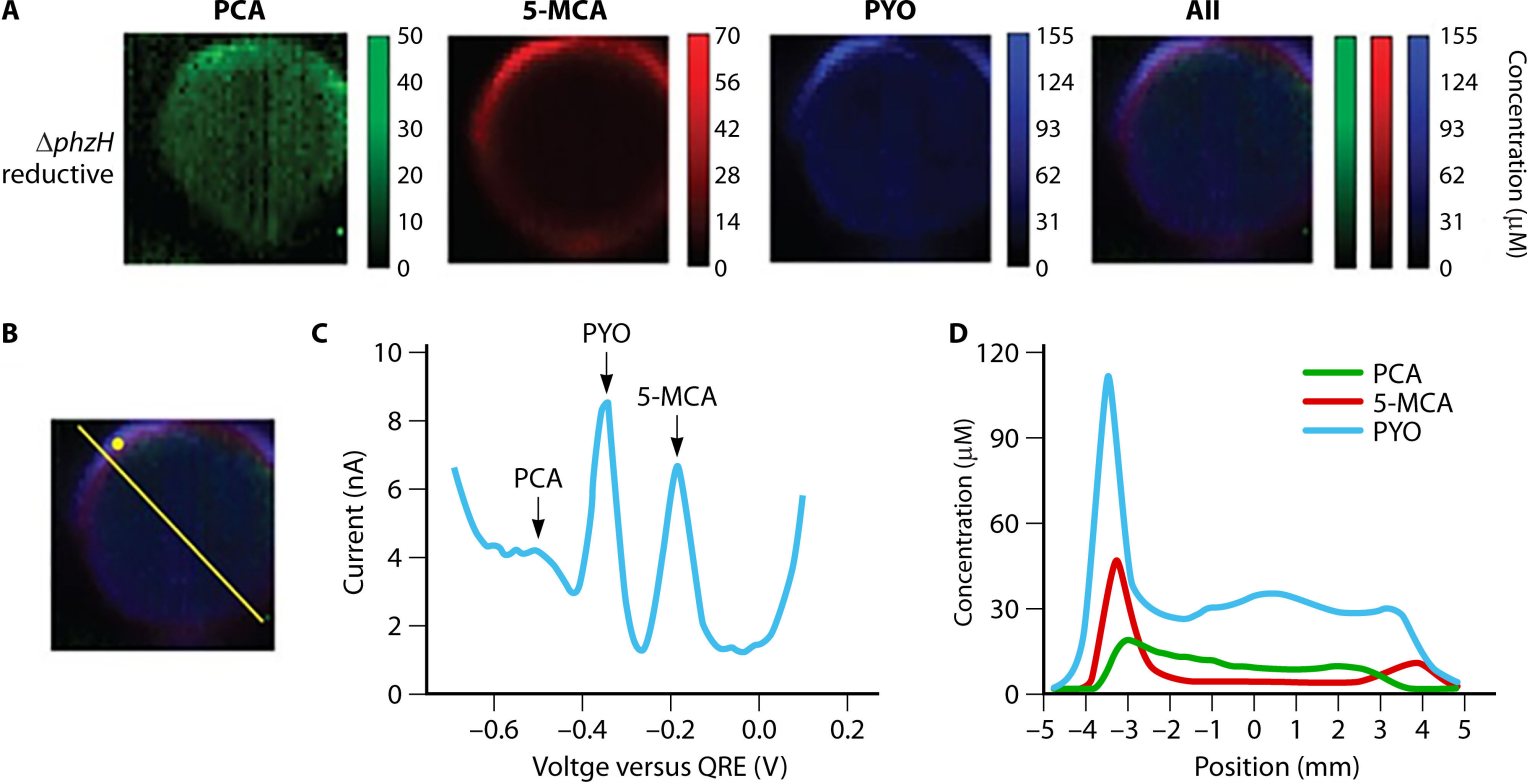
Mapping and concentration of phenazines phenazine-1-carboxylic acid (PCA), 5-methylphenazine-1-carboxylic acid (5-MCA), pyocyanin (PYO), and phenazine-1-carboxamide in a mutant *P. aeruginosa* colony biofilm using electrochemical imaging (**A**). Location and cross-section of biofilm (**B**) used for single-electrode voltammogram (**C**) and cross-section electrochemical image (**D**), respectively. Reprinted from reference (36).

The oxidized forms of PYO and phenazine carboxamide (PCN) can be retained by their ability to bind eDNA. This is supported by LC–MS of the biofilm versus the surrounding agar. In this work, the dominant form of phenazine-1-carboxylic acid (PCA) was negatively charged and was not retained but released into the surrounding environment. This localization allows redox-active phenazines to form an electron shuttling network through the ECM and provide suitable electron acceptors deep within the biofilm where oxygen is limited (153). To visualize the 2D distribution of phenazines within the biofilm, Bellin et al. (36) exploited the redox-active nature of phenazines and used electrochemical imaging of colony biofilms. Looking only at the biofilm, they found PCA throughout in the biofilm cross section, while 5-methyl-phenazine-1-carboxylic acid and PYO were concentrated at the biofilm edge (Fig. 5).

Despite PYO binding to eDNA, it also diffuses into the surrounding environment, potentially affecting neighboring cells. To understand this impact on nearby biofilms, Koley et al. applied SECM, which spatially measures redox-active compounds. This work finds that PYO secreted into the liquid media surrounding a biofilm is in a reduced state within the first few hundred microns of the biofilm but shifts to a predominantly oxidized state at farther distances. The reduced state is both antimicrobial and has the capacity to reduce insoluble Fe^3+^ to the soluble, more bioavailable, Fe^2+^ form (154), making it more accessible in the immediately surrounding environment (34).

Pyocyanin can influence cell death and subsequent release of eDNA, thus impacting biofilm structure (151, 155). Loss of either eDNA or PYO causes a similar reduction in biofilm formation, supporting the importance of PYO in the structural integrity of *P. aeruginosa* biofilms (156). Meirelles et al. find that 1-hydroxyphenazine, PCA, and PCN can all increase cell death in planktonic culture but to a lesser extent than PYO. Cell death does not occur immediately upon addition of PYO but takes place during stationary phase. Cells in nutrient-replete environments are able to counteract the lethal effects of PYO, but as nutrients are depleted, cells unable to carry out ATP synthesis become sensitive to PYO toxicity (157). Thus, as we continue to understand metabolite localization and, importantly, the co-localization, we will better understand the activity of these compounds.

## FUTURE NEEDS

In this review, we have summarized several analytical chemistry approaches that have been useful to obtain spatial chemical information of surface bacterial growth systems. We have specifically highlighted advances in our understanding of *P. aeruginosa* metabolite spatial distribution focusing on alkylquinolones, rhamnolipids, and phenazines and the chemical imaging methods used to advance this field. The approaches serve as an additional tool to complement other spatiotemporal methods such as light microscopy or spatial transcriptomics. These analytical techniques, such as confocal Raman microscopy, mass spectrometry imaging, and electrochemical imaging, have been extraordinarily useful in building 2D and 3D metabolite maps of communities. In addition to delivering spatial information, tandem use of these techniques increases the capacity to distinguish similar molecules, which often exhibit varied localization patterns. Spatial analyses have also been applied to bring light to questions such as the formation of biofouling and metabolite distribution in diseased tissues (33, 97). With the aid of improved multi-modal analyses, researchers are building better informed 2D and 3D maps of microbial species and their metabolites (158–160).

Beyond mapping the chemical variation in biofilms, identifying the drivers of biofilm heterogeneity is an area requiring further investigation. Molecules, such as alkylquinolones, are emerging as necessary initiators of biofilm heterogeneity. Alkyl quinolones promote the formation of aggregates, an example of condensed matter occurring at the biofilm level, in contrast to the well-known role of condensed matter promoting organization at the cellular level (161). Like AQs, additional molecules or groups of molecules likely also serve as drivers of bacterial community organization, shaping biofilms as they age and encounter new environments. The tools (or combined multiplex tool) do not exist yet to enable spatial understanding and characterization of the iterative processes that are occurring in surface-growing bacterial communities where (i) select genes are induced; (ii) biochemical products are produced; and (iii) the influence of these biochemical products leads to a change of the gene expression at the first step.

To move our research forward to better understand bacterial communities formed by *P. aeruginosa* and other organisms, we will need to further establish and develop means to characterize the biochemical products made by these microbes *in situ*. As resolution technology continues to improve, the mass of biomolecule produced by any single microbe is likely finite. This necessitates gaining an understanding of the biochemical output on a per-cell basis, as done by Connell et al. (37). The number of bacteria per unit area in many natural and clinical settings are likely to be significantly lower than that in laboratory *in vitro* systems. For most of the reports cited herein, the number of bacteria analyzed is not reported. This is important to understand the thresholds and at which these produced spatial cues influence their surrounding neighbors in both mono-culture and polymicrobial communities.

Some of these advances can be made by encouraging more analytical chemists to work with us on our specific research. Moreover, we need to challenge them (and ourselves) to co-adapt our standard experimental protocols to enable the measurements we desire. We need to push each other to enable multiple technologies that can visualize bacterial behavior and the associated biochemical products at a single-cell resolution in communities over time.
